# Extensive Myocardial Calcification in a Heart Transplant Patient

**DOI:** 10.36660/abc.20190146

**Published:** 2020-01

**Authors:** Sasha B. C. P. Duarte, Sandrigo Mangini, Monica S. Avila, Marcelo L. Montemor, Fernando Bacal

**Affiliations:** 1 Hospital das Clínicas da Faculdade de Medicina da Universidade de São Paulo (HCFMUSP), São Paulo, SP - Brazil

**Keywords:** Heart Transplantation/complications, Heart Valve Diseases/surgery, Renal Insufficiency/complications, Shock, Septic

**Figure 1:**
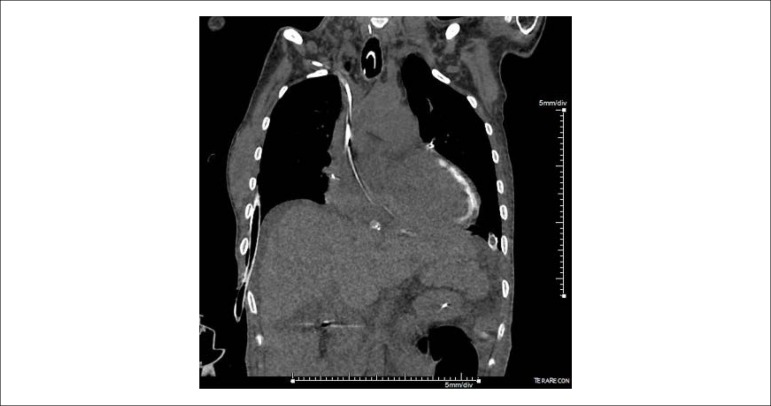
Coronary non-contrast-enhanced computed tomography scan of the chest with finding of extensive myocardial calcification in the left ventricle.

**Figure 2:**
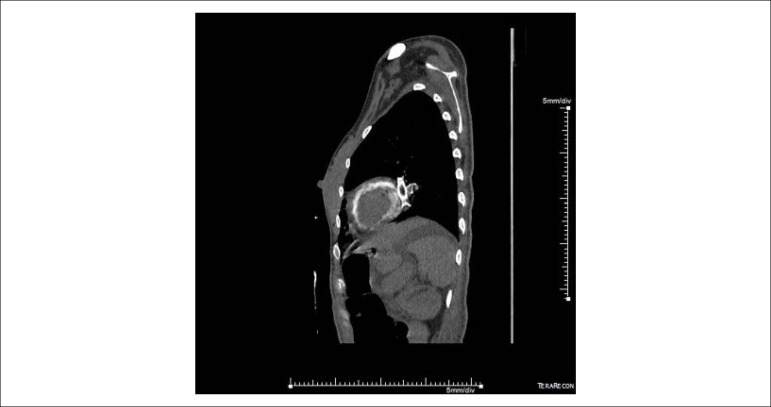
Sagittal non-contrast-enhanced computed tomography scan of the chest with finding of extensive myocardial calcification in the left ventricle.

**Figure 3:**
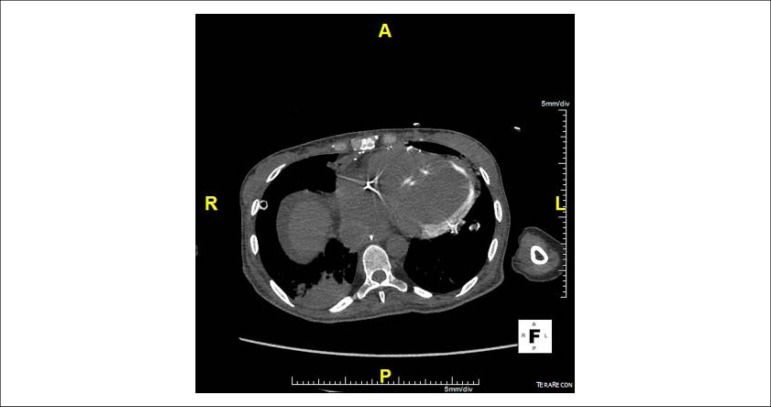
Axial non-contrast-enhanced computed tomography scan of the chest with finding of extensive myocardial calcification in the left ventricle.

A 33-year-old female patient underwent heart transplantation (Tx) for valvular heart disease, where the surgical procedure was uneventful. Post-Tx, she developed with acute graft dysfunction, acute renal failure (ARF) requiring dialysis and septic shock. Bloodstream infection confirmed by treatment for carbapenemase-producing *Klebsiella pneumoniae*. Non-contrast-enhanced computed tomography (CT) of the chest and abdomen was done for investigation of the infectious focus and distention of the abdomen and melena, with extensive left ventricular myocardial calcification (MC) not previously found in CT (Figures 1, 2 and 3). A diagnosis of cytomegalovirus (CMV) infection was also confirmed by upper digestive endoscopy findings with diffuse gastroduodenal ulcers and quantitative detection of positive CMV DNA, and the patient received ganciclovir. The patient became refractory to treatment and died.

MC is a rare complication that occurs in critically ill patients. It has various etiologies, and its pathophysiology is not completely elucidated. MC may involve mechanisms of metastatic calcification and dystrophic calcification, as presented in Table 1. It can be the cause of heart failure, sudden death, abnormalities in ventricular wall movement, arrhythmias and restrictive disease.^1^

**Table 1:** Possible myocardial calcification etiologies

Metastatic calcification (Altered serum calcium level)	Dystrophic calcification (Calcium accumulation in necrotic tissues, without hypercalcemia)
Chronic renal failure	Infections
Primary parathyroidism	Extracorporeal membrane oxygenation
Neoplasms	Inflammatory processes
Bone disturbances	Processes myocardial infarction
Medications	Myocarditis

The case demonstrates a correlation with others described in the literature, showing extensive MC in a young patient with anemia, ARF, septic shock,^2^ exposure to extracorporeal membrane oxygenation,^3^ and high mortality, with the difference being an immunosuppressed post-heart transplant patient. The true meaning of this finding and its reversibility are unknown. However, it is believed to be related to disease severity and poor prognosis, and its identification in clinical practice is important.
